# Supplementary material for: Insights on genomic profiles of drug resistance and virulence in a cohort of Leishmania infantum isolates from the Mediterranean area
Source: Parasit Vectors. 2025 Nov 29;19:28. doi: 10.1186/s13071-025-07105-2 (PMC12802309; doi:10.1186/s13071-025-07105-2)
Supplement: Supplementary file 3 — Additional file 3: Figure S1. Correlation between chromosome copy number and biomarker copy number (sub-chromosomal level). [file 13071_2025_7105_MOESM3_ESM.pdf]

Additional File 2: Figure S1. Correlation between chromosome copy number and biomarker copy number (sub-chromosomal level). The scatterplots show the Pearson correlation of chromosome copy number and biomarker copy number. Detection of three distinct correlation patterns: strong (extended H-locus and *LdMT),* moderate (*aqp1*, MSL loci) and low (*LdRos3*, LdSMT locus, METK locus, and PMM locus).

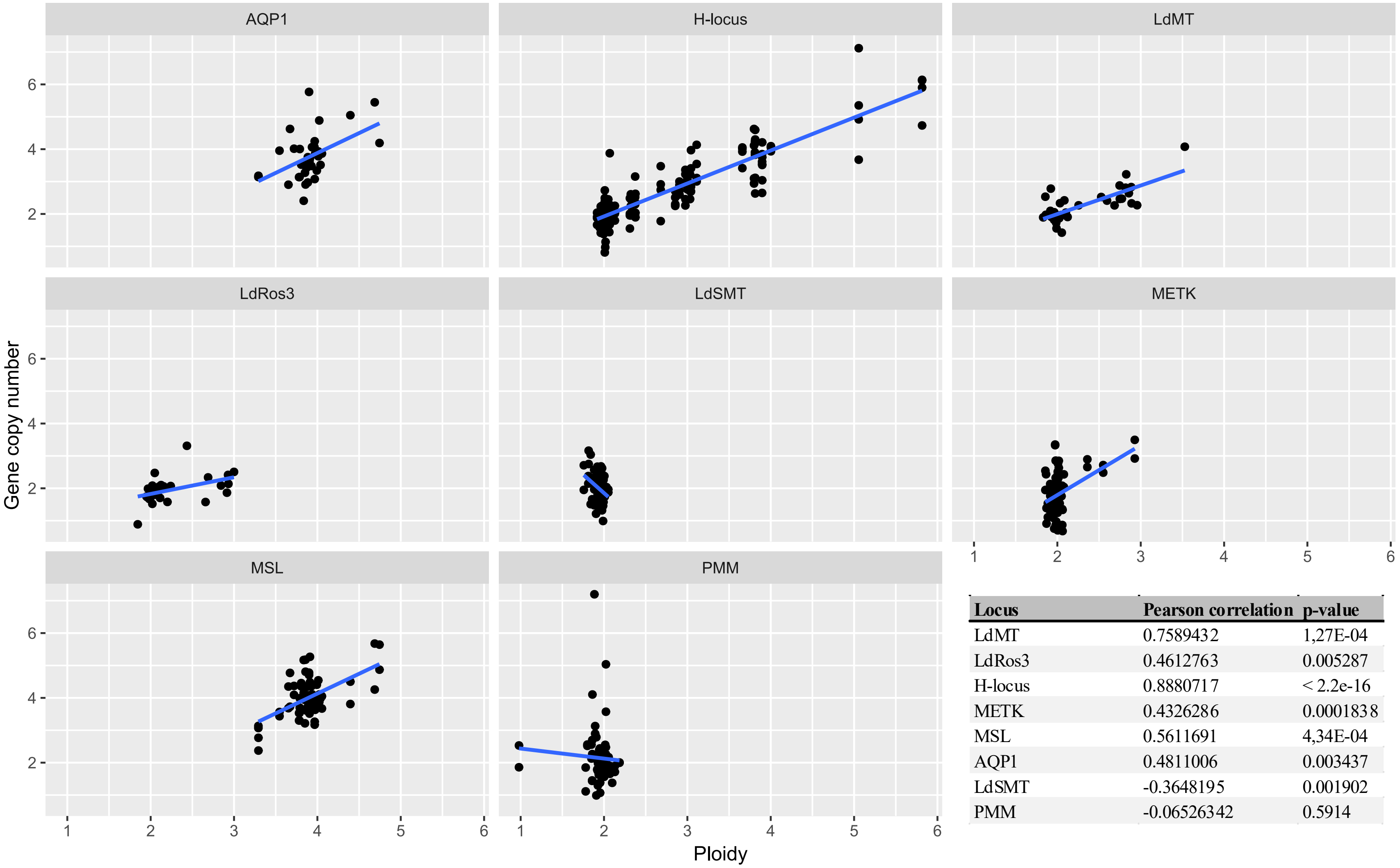

Locus	Pearson correlation	p-value
LdMT	0.7589432	1,27E-04
LdRos3	0.4612763	0.005287
H-locus	0.8880717	< 2.2e-16
METK	0.4326286	0.0001838
MSL	0.5611691	4,34E-04
AQP1	0.4811006	0.003437
LdSMT	-0.3648195	0.001902
PMM	-0.06526342	0.5914
